# Expression pattern of non-coding RNAs in non-functioning pituitary adenoma

**DOI:** 10.3389/fonc.2022.978016

**Published:** 2022-09-02

**Authors:** Soudeh Ghafouri-Fard, Arash Safarzadeh, Mehdi Akhavan-Bahabadi, Bashdar Mahmud Hussen, Mohammad Taheri, Nader Akbari Dilmaghani

**Affiliations:** ^1^ Men’s Health and Reproductive Health Research Center, Shahid Beheshti University of Medical Sciences, Tehran, Iran; ^2^ Department of Medical Genetics, School of Medicine, Shahid Beheshti University of Medical Sciences, Tehran, Iran; ^3^ University of Tehran, Tehran, Iran; ^4^ Department of Pharmacognosy, College of Pharmacy, Hawler Medical University, Erbil, Iraq; ^5^ Center of Research and Strategic Studies, Lebanese French University, Erbil, Iraq; ^6^ Urology and Nephrology Research Center, Shahid Beheshti University of Medical Sciences, Tehran, Iran; ^7^ Institute of Human Genetics, Jena University Hospital, Jena, Germany; ^8^ Skull Base Research Center, Loghman Hakim Hospital, Shahid Beheshti University of Medical Sciences, Tehran, Iran

**Keywords:** non-functioning pituitary adenoma, lncRNA, miRNA, expression, biomarker

## Abstract

Non-functioning pituitary adenoma (NFPA) is a benign tumor arising from the adenohypophyseal cells. They can be associated with symptoms arising from mass effect. Although these tumors are regarded to be benign tumors, they are associated with increased comorbidity and mortality. Several studies have indicated abnormal expression of genes in these tumors. In the current study, we have used existing methods to identify differentially expressed genes (DEGs) including DE long non-coding RNAs (DElncRNAs) and DE microRNAs (DEmiRNAs) in NFPAs compared with normal samples. Then, we have assessed the relation between these genes and important signaling pathways. Our analyses led to identification of 3131 DEGs, including 189 downregulated DEGs (such as RPS4Y1 and DDX3Y) and 2898 upregulated DEGs (such as ASB3 and DRD4), and 44 DElncRNAs, including 8 downregulated DElncRNAs (such as NUTM2B-AS1 and MALAT1) and 36 upregulated DElncRNAs (such as BCAR4 and SRD5A3-AS1). GnRH signaling pathway, Tight junction, Gap junction, Melanogenesis, DNA replication, Nucleotide excision repair, Mismatch repair and N-Glycan biosynthesis have been among dysregulated pathways in NFPAs. Taken together, our study has revealed differential expression of several genes and signaling pathways in this type of tumors.

## Introduction

Non-functioning pituitary adenoma (NFPA) is a benign tumor arising from the adenohypophyseal cells. This type of tumor is described by the lack of clinical signs of hypersecretion of hormones. Statistics show a prevalence of 7–41.3/100,000 for NFPA (1–3). The incidence of this type of tumors seems to be increased during recent years, possibly due to enhanced numbers of incidentally identified adenomas in brain imaging conducted for other purposes (4).

Eight subtypes identified for NFPA are as follow: silent gonadotroph, corticotroph, somatotroph, thyrotroph, lactotroph, plurihormonal Pit-1, null-cell, and double/triple NFPAs (5). NFPA has variable clinical manifestations ranging from asymptomatic to symptoms resulting from effects of mass on neighboring regions leading to headache, visual defect, and/or hypopituitarism (2, 6).

Although these tumors are regarded to be benign tumors from a histological point of view, they are associated with increased comorbidity and mortality (3, 7).

Recent studies have indicated abnormal expression pattern of several coding and non-coding genes in NFPA (8, 9). For instance, transcriptome analysis has shown distinct profiles in pituitary adenomas compared to the non-tumoral tissues, irrespective of the identified immunophenotype. Notably, calcium metabolism and immune-related genes are among the mostly altered genes in adenomas (9).

In the current study, we have used existing methods to identify differentially expressed genes (DEGs) including DE long non-coding RNAs (DElncRNAs) and DE microRNAs (DEmiRNAs) in NFPAs compared with normal samples. Then, we have assessed the relation between these genes and important signaling pathways.

## Methods

### Microarray data collection

We used the Gene Expression Omnibus (GEO; http://www.ncbi.nlm.nih.gov/geo/) to obtain the human expression profiles of GSE62960 (Agilent-014850 Whole Human Genome Microarray 4x44K G4112F) and GSE63357 (Affymetrix Human Genome U133 Plus 2.0 Array), which contained 28 and 25 samples, respectively. We selected 10 non-functioning pituitary adenoma samples from GSE62960 and 5 normal pituitary samples from GSE63357 for further analysis. The expression data contained both lncRNAs and mRNAs expression signatures.

### Microarray data processing, integrative meta-analysis and assessment of data quality

Processing and integration of all microarray data were performed using the R statistical programming language, the mentioned datasets have different and trendy platforms (Agilent and Affymetrix), a sensitive step in the integration of heterogeneous data is normalization (10). Batch effects (non-biological differences) were removed by applying the ComBat function from the R Package Surrogate Variable Analysis (SVA) (11). Batch effect removal was checked by PCA and boxplot. The meta-analysis outcome is a unit expression matrix (the combination of four datasets of this study). Then, we used quantile normalization method to normalize data expression matrix.

To accomplish quantile normalization, we used the preprocessCore R package. Also, we utilized ComBat function based on its description in sva package (ComBat permits adjustment of batch effects in datasets where the batch covariate is known (12)) and bioconductor (The sva package can be utilized to eliminate artifacts by three methods: (1) recognizing and appraising surrogate variables for unknown sources of variation in high-throughput experiments (13), (2) directly eliminating identified batch effects using ComBat (12) and (3) removing batch effects with known control probes. We used this function after merging two datasets. This method has been used in recent publications as well (14).

### Analysis of differentially expressed lncRNAs and mRNAs

The Limma package in R language (15) was used to obtain DEGs and DElncRNAs between NFPA and normal samples. Furthermore, we used Bonferroni in the multtest package to adjust the *P* value into the FDR. We used the FDR < 0.05 and |log_2_ FC| > 1 as the cutoff criteria for DEGs and DElncRNAs. Then we identified DElncRNAs using HUGO gene nomenclature committee.

### Two-way clustering of DEGs and DElncRNAs

The gene expression parameters of substantial differentially expressed genes and lncRNAs were obtained. Then, the pheatmap package in R language (version 1.0.12) (16) was used to conduct the two-way clustering based on the Euclidean distance.

### Gene ontology (GO) enrichment analyses

In order to find the function of the obtained considerably downregulated and upregulated DEGs, we performed Gene Ontology (GO) enrichment analysis using the clusterProfiler R package (17). We set *p*-value < 0.05 as the thresholds of the functional categories.

### Kyoto encyclopedia of genes and genomes (KEGG) pathway analysis

KEGG pathway analysis of considerably downregulated and upregulated DEGs was performed to find the potential function of these genes contributing to the pathways based on the KEGG database (18).

### Constructing the ceRNA network

We built a ceRNA network through the following steps: 1) Searching the miR2Disease database (http://watson.compbio.iupui.edu:8080/miR2Disease/index.jsp) (19) for the pituitary adenoma (PA)-related miRNAs using the keyword “Pituitary Adenoma”. 2) Using miRcode (http://www.mircode.org/) for assessment of interaction between lncRNAs and miRNAs based on the PA-related miRNAs; 2) Application of miRDB (http://www.mirdb.org/) (20), TargetScan (http://www.targetscan.org/) (21) and miRWalk (http://129.206.7.150/)  (22) for prediction of miRNAs-targeted mRNAs; 3) Finding the intersections of the differentially expressed lncRNAs and mRNAs, and establishment of lncRNA/mRNA/miRNA ceRNA network using Cytoscape v3.0 (23); and 4) we used cytohubba (24) to detect 15 hub genes with best degree in ceRNA network.

### Survival analysis

GEPIA (25) was used to depict survival curves according to prognostic value of top 10 genes with best degree in ceRNA network. The clinical data for patients with low grade glioma was obtained from TCGA. The TCGA-LGG data (https://portal.gdc.cancer.gov/) included 515 primary solid tumor samples. This analysis was done on June 9, 2022.

Kaplan–Meier curves were depicted for evaluation of univariate survival. P-values less than 0.05 were considered statistically significant.

## Results

### Dataset quality assessment

**Figure 1:**
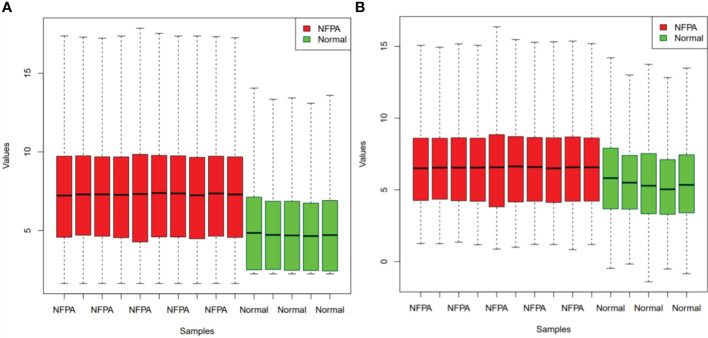
Boxplots for the data before **(A)** and after **(B)** batch effect removal. Red boxes indicate NFPA samples and green boxes show healthy samples.

**Figure 1** demonstrates the boxplot of raw data before and after batch effect removal. These boxplots indicates that the quality of the expression data was reliable.

**Figure 2:**
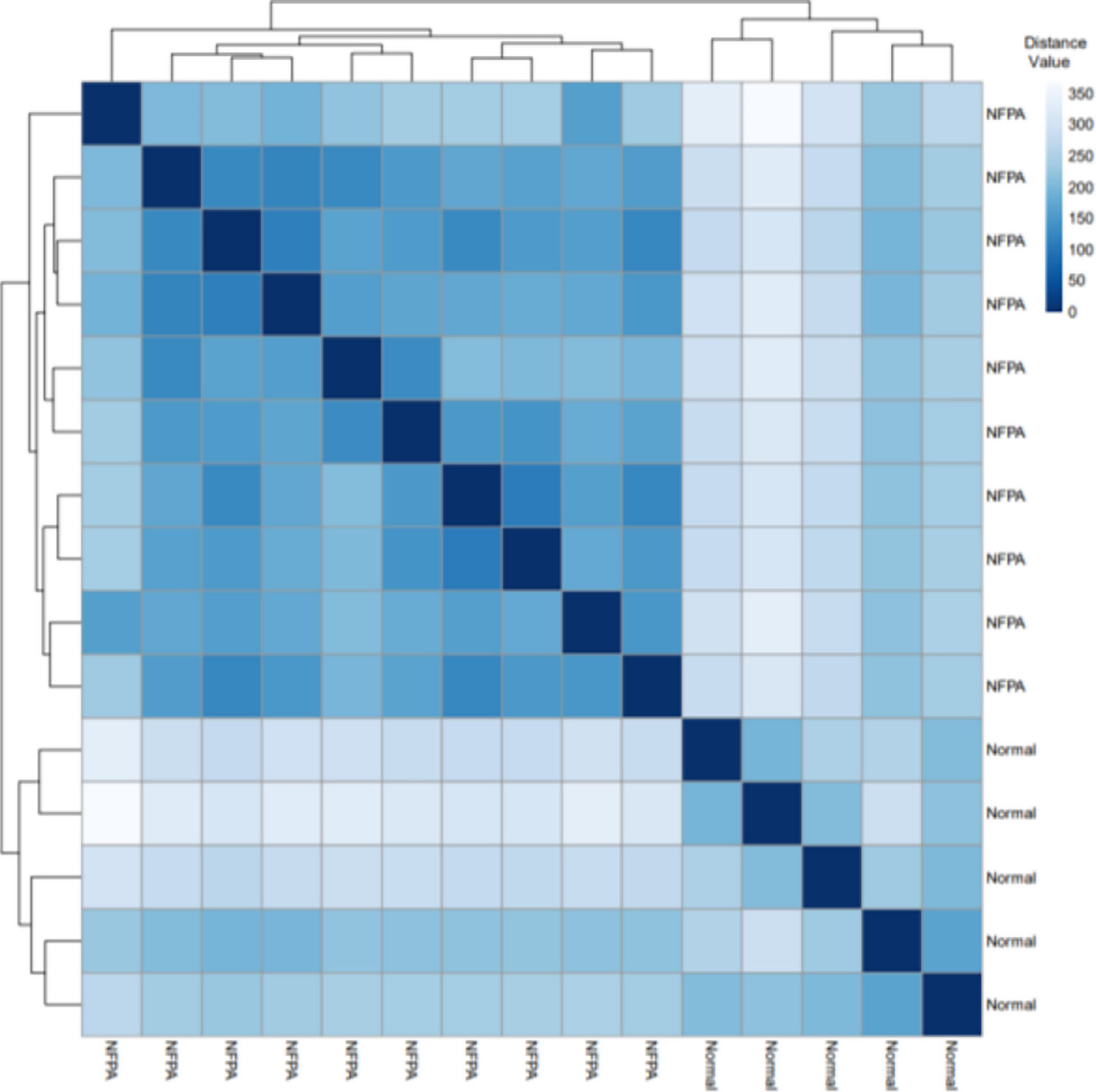
The Euclidean distances between the samples. Based on the Euclidean distance, hierarchical clustering between the samples has been established; the distance values between samples are shown.

**Figure 2** displays the Euclidean distances between the samples after batch effect removal. Tumor and healthy samples were divided into two groups and put into two clusters.

**Figure 3:**
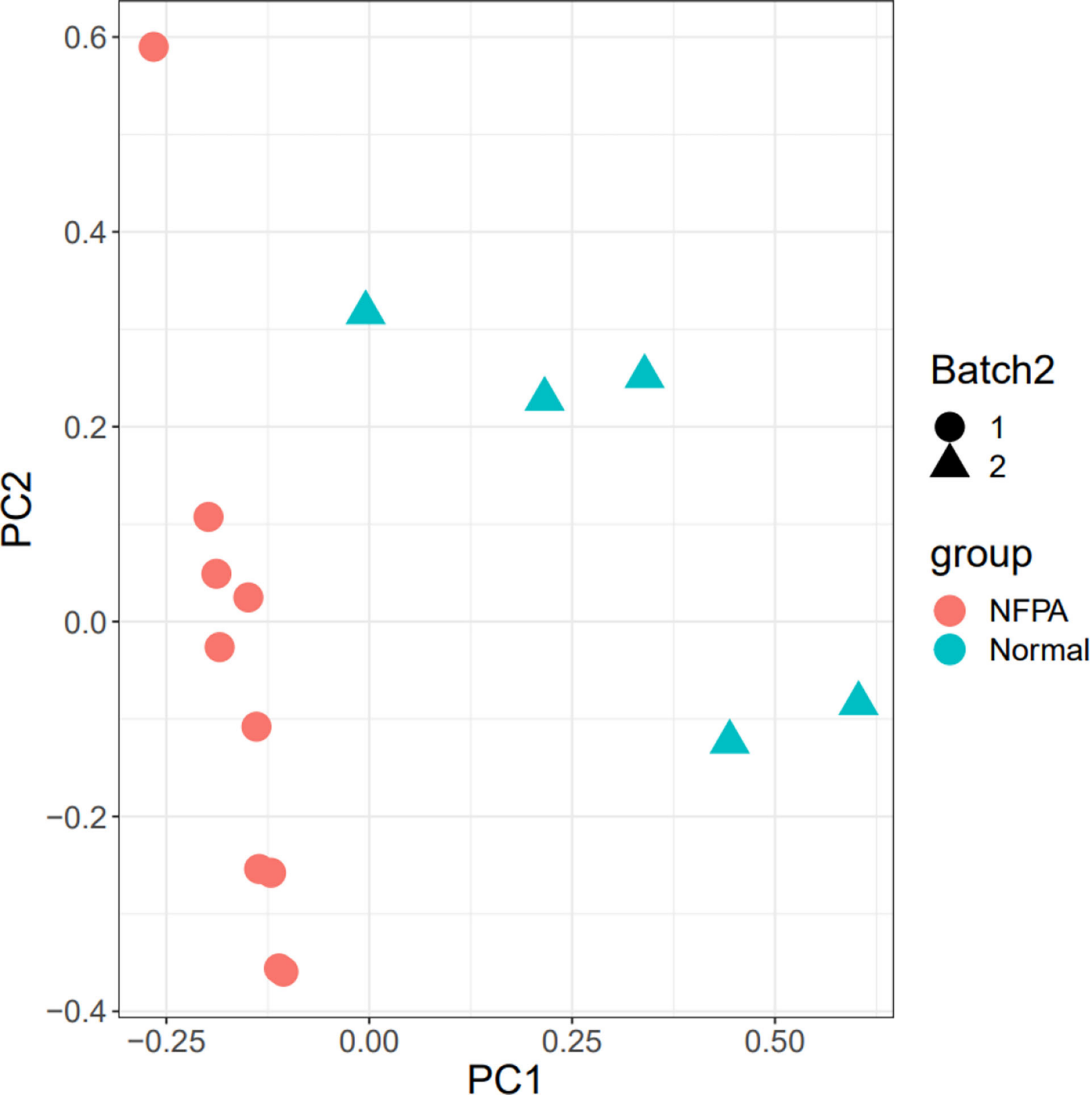
PCA plot. The Batch implies that the data includes two platforms. Also, healthy and tumor samples were divided into two groups.

The 15 samples are displayed in the 2D plane covered by their first two principal components in the PCA plot (PC1 and PC2) (**Figure 3**). This plot shows the good relative variance between the NFPA and normal samples.

### DEGs analysis

According to analyses of the microarray data between NFPA and normal samples by limma, we obtained 3131 DEGs, including 189 downregulated DEGs (such as RPS4Y1 and DDX3Y) and 2898 upregulated DEGs (such as ASB3 and DRD4), and 44 DElncRNAs, including 8 downregulated DElncRNAs (such as NUTM2B-AS1 and MALAT1) and 36 upregulated DElncRNAs (such as BCAR4 and SRD5A3-AS1). **Table 1** lists the top 10 markedly downregulated and upregulated DEGs.

**Table 1:** The top 10 up- and downregulated DEGs between NFPA and normal samples.

Down-regulated			Up-regulated		
DEG	Log FC	Adjusted *P* value	DEG	Log FC	Adjusted *P* value
RPS4Y1 DDX3Y POMC SFPQ KDM5D CRYAB TSHB PAX6 SCUBE3 CGA	-6.247364 -5.227572 -4.809255 -4.447223 -4.205645 -4.043984 -3.925312 -3.889940 -3.822918 -3.685164	0.033317521 0.026558724 0.020522011 0.016269745 0.044775414 0.006188775 0.003632825 0.002707552 0.003706830 0.013048913	ASB3 DRD4 LOC646626 LOC100130331 HIST1H2BO RRN3P3 SNORA78 EGLN2 HIST1H3C TACR2	9.711828 9.339609 8.928223 8.411643 8.230407 8.210715 8.092251 7.175348 7.109154 7.056791	0.002707552 0.003678029 0.002707552 0.002707552 0.002765520 0.002707552 0.002859834 0.002707552 0.002795396 0.003065875

**Table 2** lists the markedly downregulated and upregulated lncRNAs.

**Table 2:** The significantly up- and downregulated DElncRNAs between NFPA and normal samples.

Down-regulated			Up-regulated		
DEG	Log FC	Adjusted *P* value	DEG	Log FC	Adjusted *P* value
NUTM2B-AS1 MALAT1 LINC00641 RNF157-AS1 LINC00899 MIR31HG LINC00844 SPANXA2-OT1	-2.536098 -2.070053 -1.981903 -1.707092 -1.649536 -1.624021 -1.348868 -1.287386	0.01392066 0.04329433 0.04098596 0.04113265 0.0258828 0.006210766 0.04505837 0.04430635	LINC00174 ARIH2OS SRD5A3-AS1 PXN-AS1 LIFR-AS1 URB1-AS1 BCAR4 LINC00886 NEBL-AS1 ATP6V0E2-AS1 LINC01003 SCAMP1-AS1 LOH12CR2 RAP2C-AS1 APTR LINC00667 EPB41L4A-AS1 TP53TG1 ST7-AS1 FGD5-AS1 LINC00842 TRAM2-AS1 RBM26-AS1 WWC2-AS2 UMODL1-AS1 HEIH IDH1-AS1 GSN-AS1 ADORA2A-AS1 GAS5 DUBR C2orf27A MAPKAPK5-AS1 HOXA-AS3 ARHGAP5-AS1 TRAF3IP2-AS1	7.536962 4.488056 4.34513 4.322818 4.262626 3.614134 3.22296 3.089068 2.937485 2.856039 2.855841 2.807056 2.751739 2.732042 2.724516 2.673574 2.672387 2.642199 2.60673 2.541793 2.468281 2.380206 2.318657 2.278828 2.172951 2.148043 1.98541 1.926714 1.917936 1.72566 1.513353 1.48974 1.360005 1.262868 1.240983 1.204049	0.003117761 0.004377148 0.002795396 0.008798058 0.007013894 0.01095202 0.002795396 0.02782602 0.04471735 0.01387096 0.004992219 0.01915909 0.02594852 0.02622078 0.01066791 0.0145873 0.00749758 0.009631829 0.04587292 0.02155033 0.01051766 0.04869672 0.03268624 0.02880773 0.04606644 0.04100989 0.01740445 0.02052201 0.03131871 0.01252509 0.01594373 0.04095487 0.01369419 0.02831569 0.04245685 0.03087259

**Figure 4:**
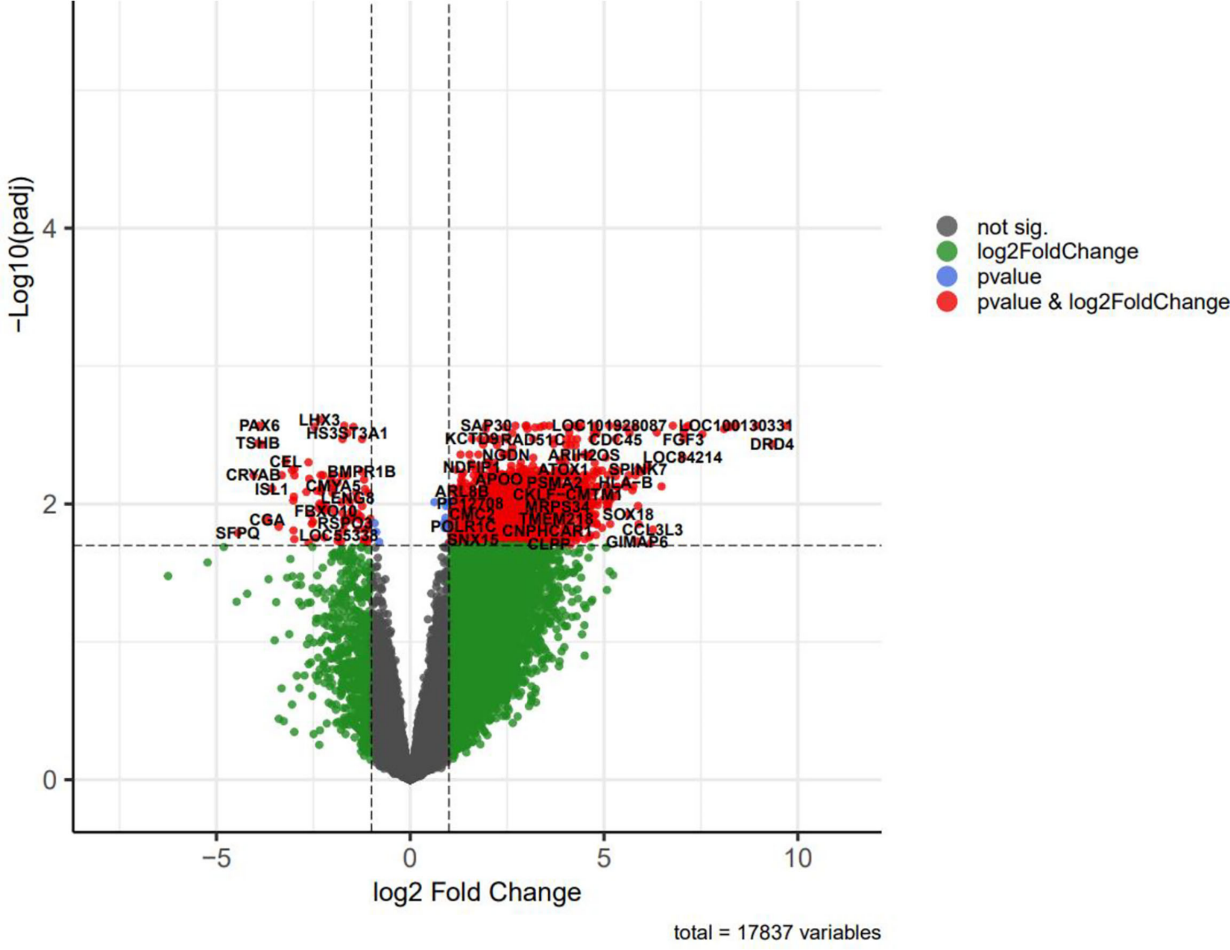
The volcano plot of differentially expressed genes (DEGs); horizontal axis, log_2_(FC); vertical axis, -log10 (adjusted P value).

Volcano plots were depicted to visualize and assess variation (or reproducibility) of lncRNA and mRNA expressions between NFPA and normal samples (**Figure 4**). Some of the differentially expressed genes included in the tables were displayed in this plot.

**Figure 5:**
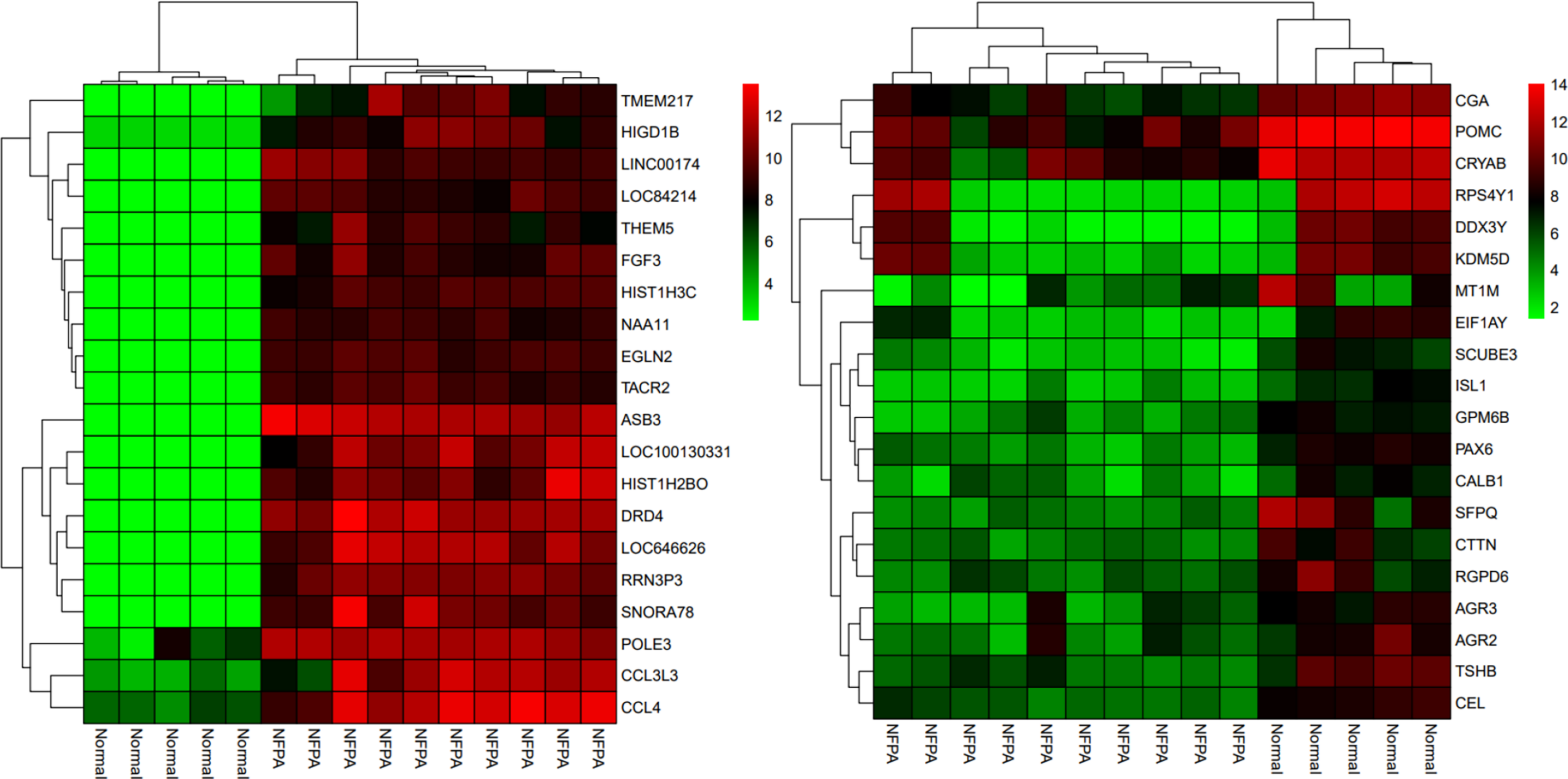
The two-way clustering of top 20 DEGs between NFPA samples and normal tissue samples; horizontal axis, the samples; vertical axis, DEGs between Normal tissues samples and NFPA samples.

**Figure 6:**
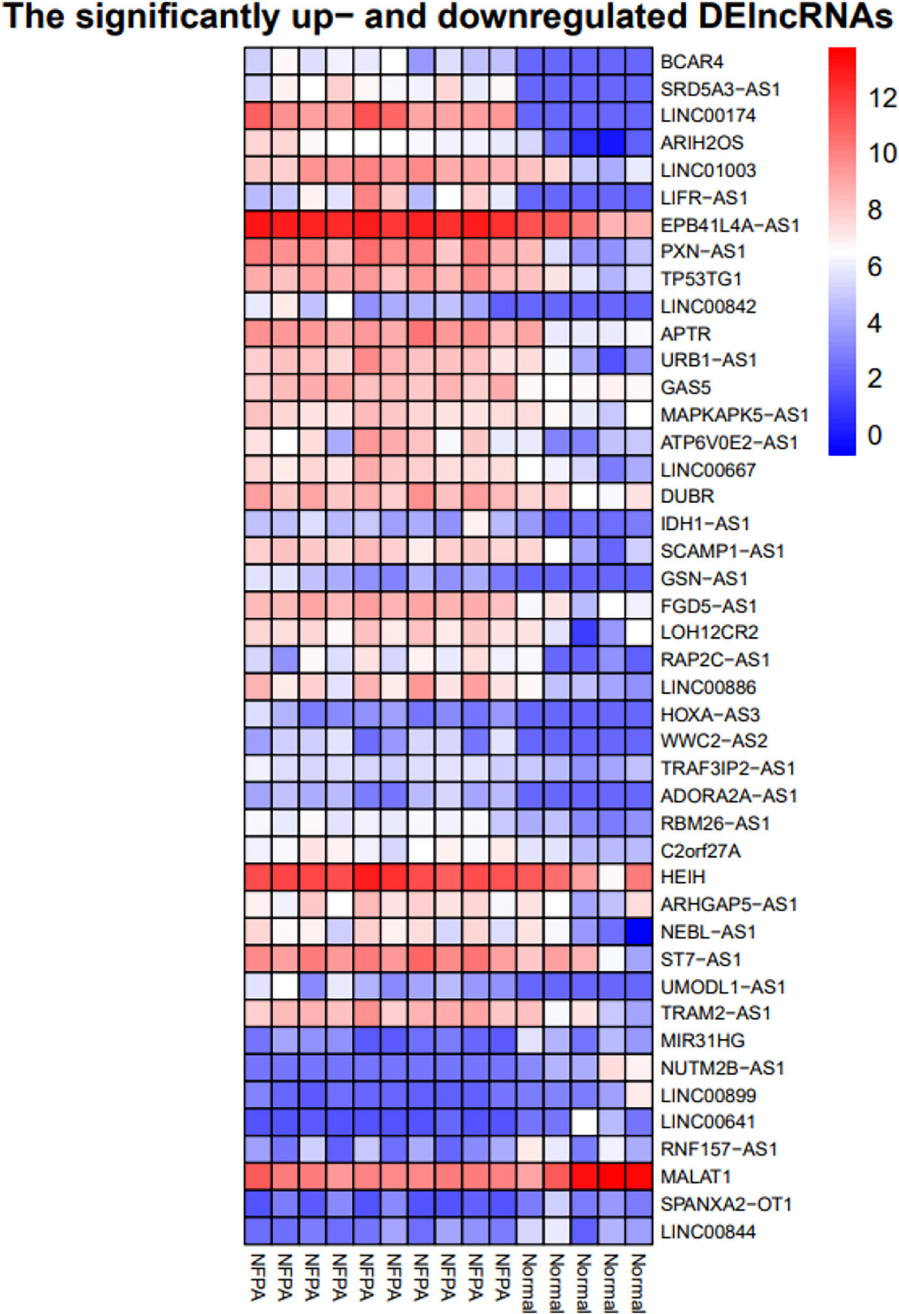
Heatmap of differentially expressed lncRNAs. The horizontal axis shows the names of 15 samples. The vertical axis presents the lncRNAs names.

Besides, the two-way clustering showed that lncRNAs and mRNAs expression pattern between PA and healthy controls was distinctive (**Figure 5**). Also, a heatmap depicts the expression of these DElncRNAs (**Figure 6**).

### GO enrichment analysis of DEGs

The noticeably DEGs were enriched in 3171 GO terms. We used Clusterprofiler package to perform analysis. in GO functional enrichment analysis, 25 GO entries satisfy Adjusted P value less than 0.05, most of which are biological processes, followed by cellular component and molecular function. The first 25 entries are integral component of endoplasmic reticulum membrane, intrinsic component of endoplasmic reticulum membrane, extracellular exosome, extracellular organelle, extracellular vesicle, mitochondrial inner membrane, mitochondrial envelope, mitochondrial membrane, carbohydrate binding, antigen binding, hormone activity, G protein-coupled receptor activity, diencephalon development, endocrine system development, cell fate specification, small molecule metabolic process, immune response, sensory organ development, immune effector process, cell fate commitment, adaptive immune response, forebrain development, pancreas development, cell differentiation in spinal cord and response to interferon-gamma. **Figures S1** and **S2** show the barplots of function enrichment analyses and GO enrichment analysis of DEGs, respectively.

### Pathway analysis

**Figure 7:**
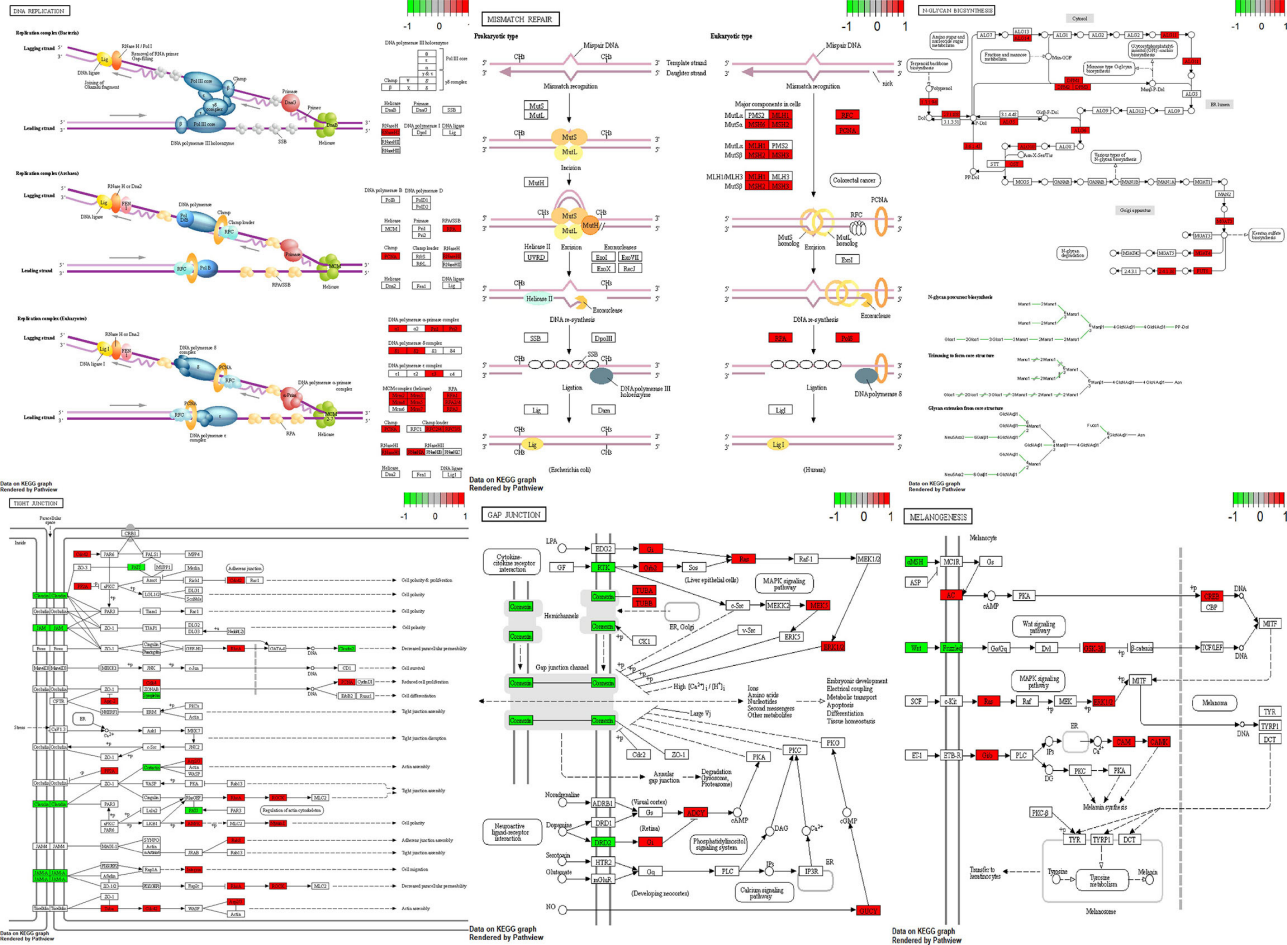
Visualization of pathways. Green boxes are downregulated genes and red boxes are upregulated genes.

Using Pathview (26) and gage (27) packages in R, KEGG pathways analysis of 189 downregulated and 2898 upregulated DEGs were conducted to detect the potential functional genes (**Table 3** and **Figure 7**).

**Table 3:** Up-regulated and down-regulated pathways.

Down-regulated		Up-regulated	
Pathway	P value	DEG	P value
GnRH signaling pathway Tight junction Gap junction Melanogenesis	0.01958461 0.02421712 0.04006672 0.04636715	DNA replication Nucleotide excision repair Mismatch repair N-Glycan biosynthesis	0.01894638 0.03886668 0.04046353 0.04360584

### ceRNA network construction in NFPA

**Figure 8:**
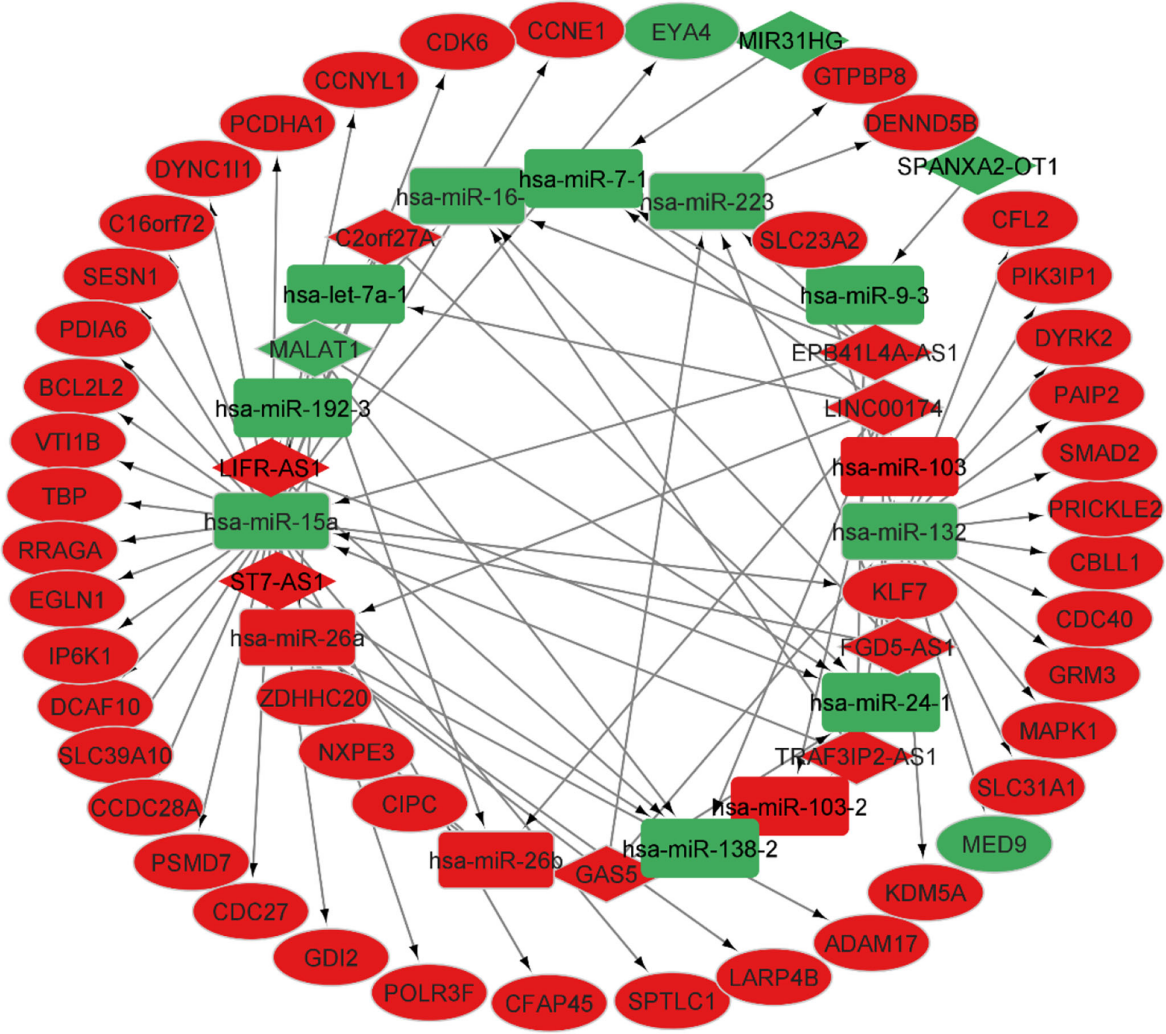
CeRNA network in NFPA. Red nodes show a high level of expression, while green nodes show a low level of expression. Ellipses represent protein-coding genes; round rectangles represent miRNAs; diamonds show lncRNAs; gray edges indicate lncRNA-miRNA-mRNA interactions.

**Figure 9:**
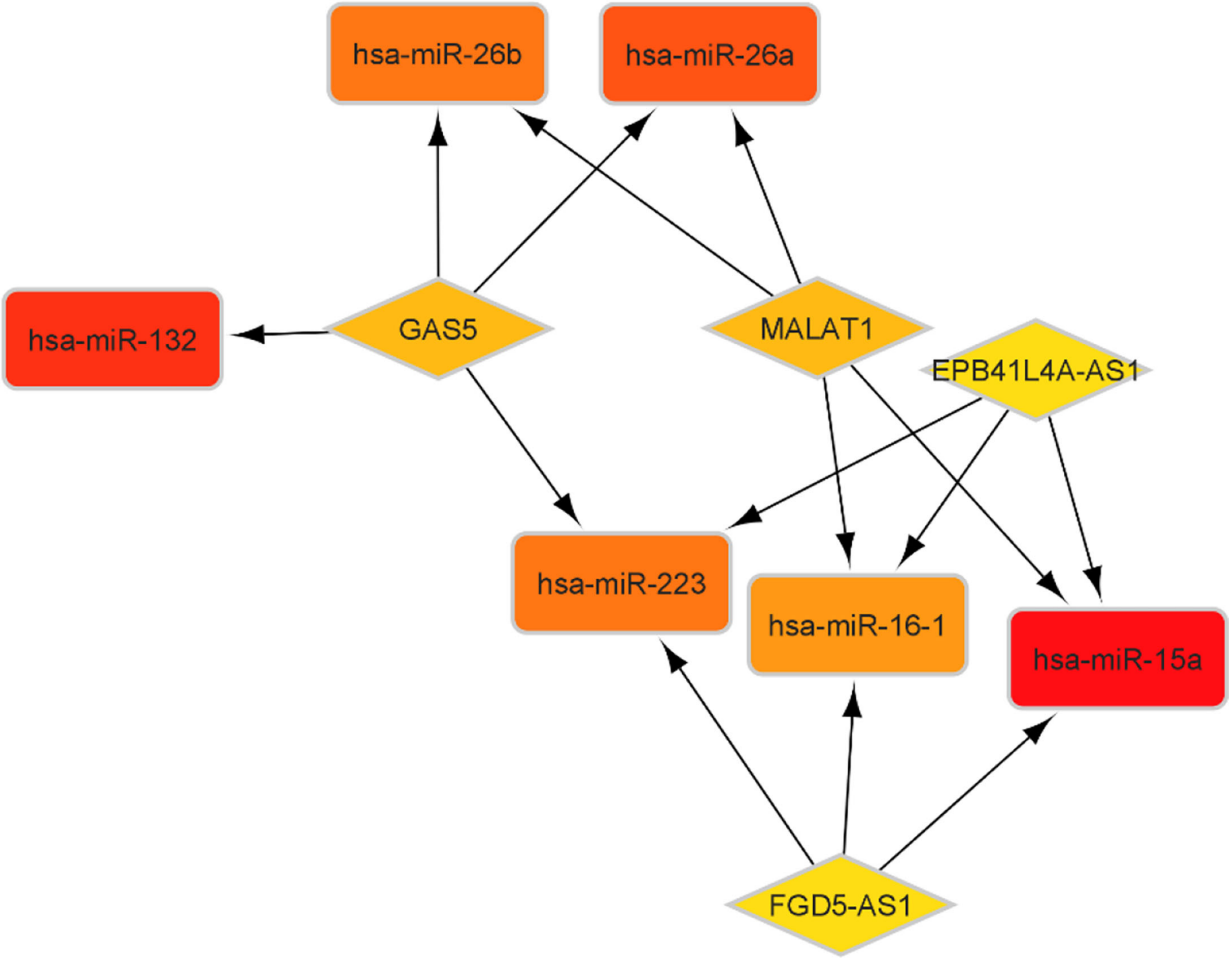
Top 10 genes with best degree in ceRNA network.

According to the miR2Disease database, we identified 26 PA-related miRNAs. Then, miRcode was used to assess interaction between lncRNAs and miRNAs. This step showed that 14 of 26 PA-specific miRNAs may target to the 11 of 44 lncRNAs (**Table 4**). Subsequently, miRDB, TargetScan and miRWalk were used for prediction of these 12 miRNA-targeted mRNAs to find the relationship between miRNAs and mRNAs. Only 5 PA-specific miRNAs were found that might target 51 of the 3131 NFPA-specific mRNAs (**Table 5**). miRNA-targeted mRNAs were excluded in the case that they were not detected in DERNAs. Accordingly, Cytoscape 3.9 was used for construction of lncRNA-miRNA-mRNA ceRNA network. A total of 11 lncRNAs, 51 mRNAs, and 14 miRNAs were included in the ceRNA network (**Figure 8**). Finally, we computed nodes degrees and displayed 10 hub genes in the network using cytohubba app (24) (**Figure 9**). We found has-miR-15a, has-miR-132, has-miR-26a, has-miR-26b, has-miR-223, has-miR-16-1, MALAT1, GAS5, EPB41L4A-AS1 and FGD5-AS1 as 10 hub genes in ceRNA network.

**Table 4:** The MiRcode database revealed interactions between 12 DElncRNAs and 14 DEmiRNAs.

lncRNA	miRNA
**MIR31HG, LINC00174, EPB41L4A-AS1**	hsa-miR-7-1
**MALAT1, EPB41L4A-AS1, C2orf27A, TRAF3IP2-AS1, FGD5-AS1**	hsa-miR-16-1
**MALAT1, EPB41L4A-AS1, C2orf27A, TRAF3IP2-AS1, FGD5-AS1**	hsa-miR-15a
**MALAT1, LIFR-AS1, ST7-AS1**	hsa-miR-192-3
**MALAT1, LINC00174, GAS5**	hsa-miR-26a
**MALAT1, LINC00174, GAS5**	hsa-miR-26b
**MALAT1, LINC00174, LIFR-AS1, GAS5, C2orf27A, TRAF3IP2-AS, FGD5-AS1**	hsa-miR-24-1
**MALAT1, LINC00174, LIFR-AS1, GAS5, ST7-AS1**	hsa-miR-138-2
**SPANXA2-OT1, EPB41L4A-AS1, TRAF3IP2-AS1**	hsa-miR-9-3
**LINC00174, C2orf27A**	hsa-let-7a-1
**LINC00174, TRAF3IP2-AS1**	hsa-miR-103
**LINC00174, TRAF3IP2-AS1**	hsa-miR-103-2
**EPB41L4A-AS1, GAS5, FGD5-AS1**	hsa-miR-223
**GAS5**	hsa-miR-132

**Table 5:** miRWalk, miRDB and TargetScan databases revealed interactions between 5 DEmiRNAs and 51 DEmRNAs.

miRNA	mRNA
**hsa-miR-15a**	SPTLC1, CFAP45, POLR3F, GDI2, CDC27, PSMD7, CCDC28A, SLC39A10, DCAF10, IP6K1, EGLN1, RRAGA, TBP, VTI1B, BCL2L2, PDIA6, SESN1, C16orf72, DYNC1I1, PCDHA1, CCNYL1, CDK6, CCNE1, KLF7, EYA4
**hsa-miR-26a**	ADAM17, LARP4B, ZDHHC20, NXPE3, CIPC
**hsa-miR-26b**	ZDHHC20, NXPE3, CIPC
**hsa-miR-223**	GTPBP8, SLC23A2, DENND5B,
**hsa-miR-132**	CFL2, PIK3IP1, DYRK2, PAIP2, SMAD2, PRICKLE2, CBLL1, CDC40, GRM3, MAPK1, SLC31A1, MED9, SLC23A2, KDM5A, KLF7

### Survival analysis

**Figure 10:**
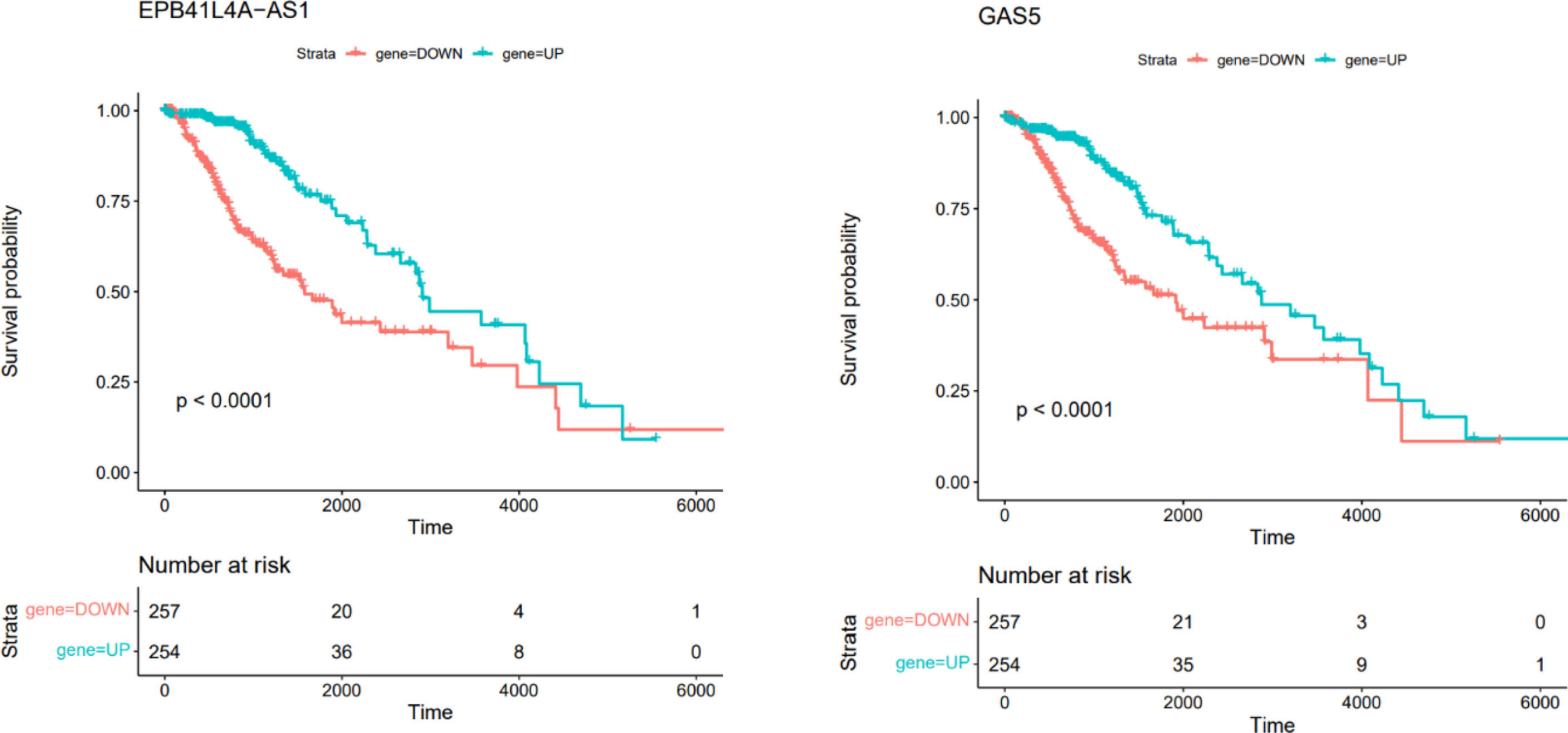
Kaplan–Meier survival curves of DElncRNAs associated with overall survival of patients with low grade glioma.

In this section, we retrieved RNA-seq data of brain low grade glioma. Survival analysis was performed based on Kaplan-Meier curve analyses using Survival package in R. We performed the survival analysis based on the hub genes in ceRNA network. The difference was regarded significant with log-rank *P* < 0.05. This analysis showed that EPB41L4A-AS1 and GAS5 were correlated with low survival time in patients with brain low grade glioma (**Figure 10**).

## Discussion

The current study aimed to identify DEGs between NFPAs and normal samples and find the importance of these genes in the pathoetiology of this disorder. Our analyses led to identification of 3131 DEGs, including 189 downregulated DEGs (such as RPS4Y1 and DDX3Y) and 2898 upregulated DEGs (such as ASB3 and DRD4). RPS4Y1 and DDX3Y have been among downregulated genes in 12 cancers in a recent whole transcriptome analysis (28). The dopamine receptor DRD4 is also among important gens in the carcinogenic processes (29).

Moreover, we found 44 DElncRNAs, including 8 downregulated DElncRNAs (such as NUTM2B-AS1 and MALAT1) and 36 upregulated DElncRNAs (such as BCAR4 and SRD5A3-AS1). Notably, MALAT1 is commonly regarded as an oncogene in the carcinogenic processes. However, some reports have suggested a tumor-suppressing effect for MALAT1 (30, 31). It seems that MALAT1 exerts an anti-cancer effect in NFPAs. In addition, NUTM2B‐AS1 has been among up‐regulated lncRNAs in hepatocellular carcinoma (HCC) whose expressions have been associated with poor prognosis of affected persons. This lncRNA has also been found to participate in the construction of ceRNA network in this type of cancer (32). Thus, the aforementioned results indicate distinct role of some lncRNAs in the pathogenesis of different types of cancers. BCAR4 has been shown to exert an oncogenic role in breast cancer inducing endocrine resistance in these cells (33). SRD5A3-AS1 is transcribed from the antisense region of SRD5A3, a gene that induces tumor growth and is associated with poor survival of HCC (34).

Hsa-miR-15a, hsa-miR-26a, hsa-miR-26b, hsa-miR-223 and hsa-miR-132 are related miRNAs with these lncRNAs. miR-26a and miR-26b are two tumor suppressor miRNAs in colorectal cancer that can suppress aggressive behavior of cancer cells through regulating FUT4 (35). In addition, miR-15a has been shown to target several oncogenes, such as BCL2, MCL1, CCND1, and WNT3A. This miRNA has been reported to be down-regulated in chronic lymphocytic lymphoma, pituitary adenomas, and prostate carcinoma (36). miR-132 and miR-223 have been shown to regulate positive feedback circuit through regulation of FOXO3a (37).

GnRH signaling pathway, Tight junction, Gap junction, Melanogenesis, DNA replication, Nucleotide excision repair, Mismatch repair and N-Glycan biosynthesis have been among dysregulated pathways in NFPAs. Thus, DNA repair systems are implicated in the pathogenesis of NFPAs.

Then, we constructed a ceRNA network which included 11 lncRNAs, 51 mRNAs, and 14 miRNAs. This ceRNA network not only represents complicated pathoetiology of NFPAs, but also provides candidates for targeted therapy of this kind of tumor.

Two DElncRNAs, including EPB41L4A-AS1 and GAS5 have been associated with survival time of patients with brain tumors. Although the association between expression pattern of these lncRNAs and mortality or morbidity of patients with NFPAs has not been investigated yet, this finding suggests the importance of these lncRNAs in this regard. GAS5 lncRNA is mainly regarded as a tumor suppressor in human cancers. This lncRNA is down-regulated in several kinds of cancer, regulating cellular processes such as cell proliferation, apoptosis and invasion. Down-regulation of GAS5 expression is associated with higher ability of proliferation and poor prognosis in some malignancies (38). Association between expression of EPB41L4A-AS1 and survival of patients has been less studied. A single study in colorectal cancer has revealed an oncogenic role for this lncRNA and indicated it as a regulator of Rho/ROCK pathway (39).

Taken together, our study has revealed differential expression of several genes and signaling pathways in this type of tumors. Some of the identified DE genes in NFPAs are predicted to exert a specific role in this type of tumor. Others have common effects in the regulation of cell proliferation in several types of cancers.

## Data availability statement

The original contributions presented in the study are included in the article/**Supplementary Material**. Further inquiries can be directed to the corresponding authors.

## Author contributions

SG-F wrote the draft and revised it. MT and MA-B designed and supervised the study. BH, AS, and ND collected the data and designed the figures and tables. AS performed the bioinformatic analysis. All the authors read the submitted version and approved it.

## Acknowledgments

The authors would like to thank the clinical Research Development Unit (CRDU) of Loghman Hakim Hospital, Shahid Beheshti University of Medical Sciences, Tehran, Iran for their support, cooperation and assistance throughout the period of study (Grant Number 43002285).

## Conflict of interest

The authors declare that the research was conducted in the absence of any commercial or financial relationships that could be construed as a potential conflict of interest.

## Publisher’s note

All claims expressed in this article are solely those of the authors and do not necessarily represent those of their affiliated organizations, or those of the publisher, the editors and the reviewers. Any product that may be evaluated in this article, or claim that may be made by its manufacturer, is not guaranteed or endorsed by the publisher.
