# Supplementary material for: Expression pattern of non-coding RNAs in non-functioning pituitary adenoma
Source: Front Oncol. 2022 Sep 2;12:978016. doi: 10.3389/fonc.2022.978016 (PMC9478794; doi:10.3389/fonc.2022.978016)
Supplement: Supplementary file 1 [file DataSheet_1.docx]

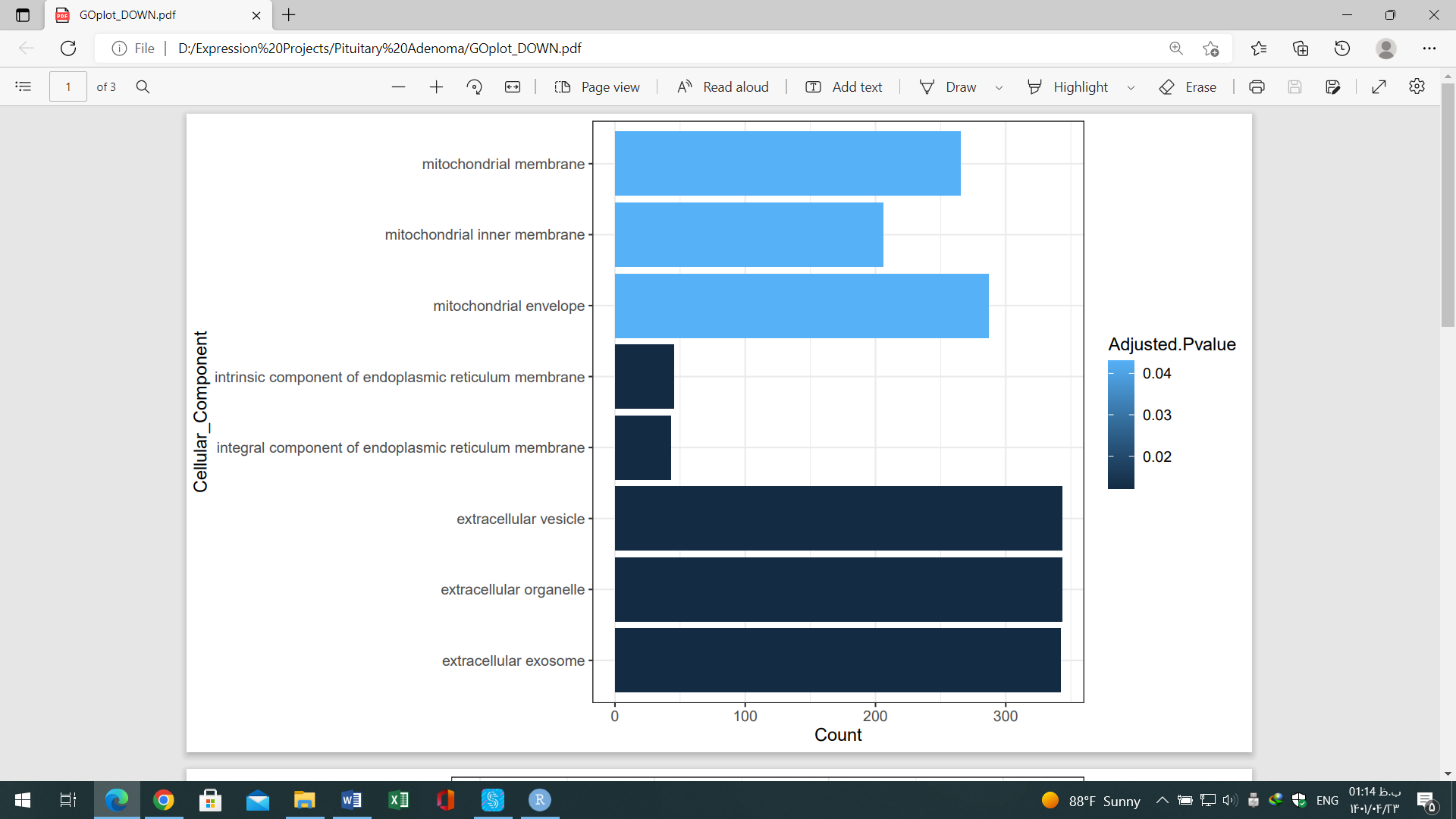


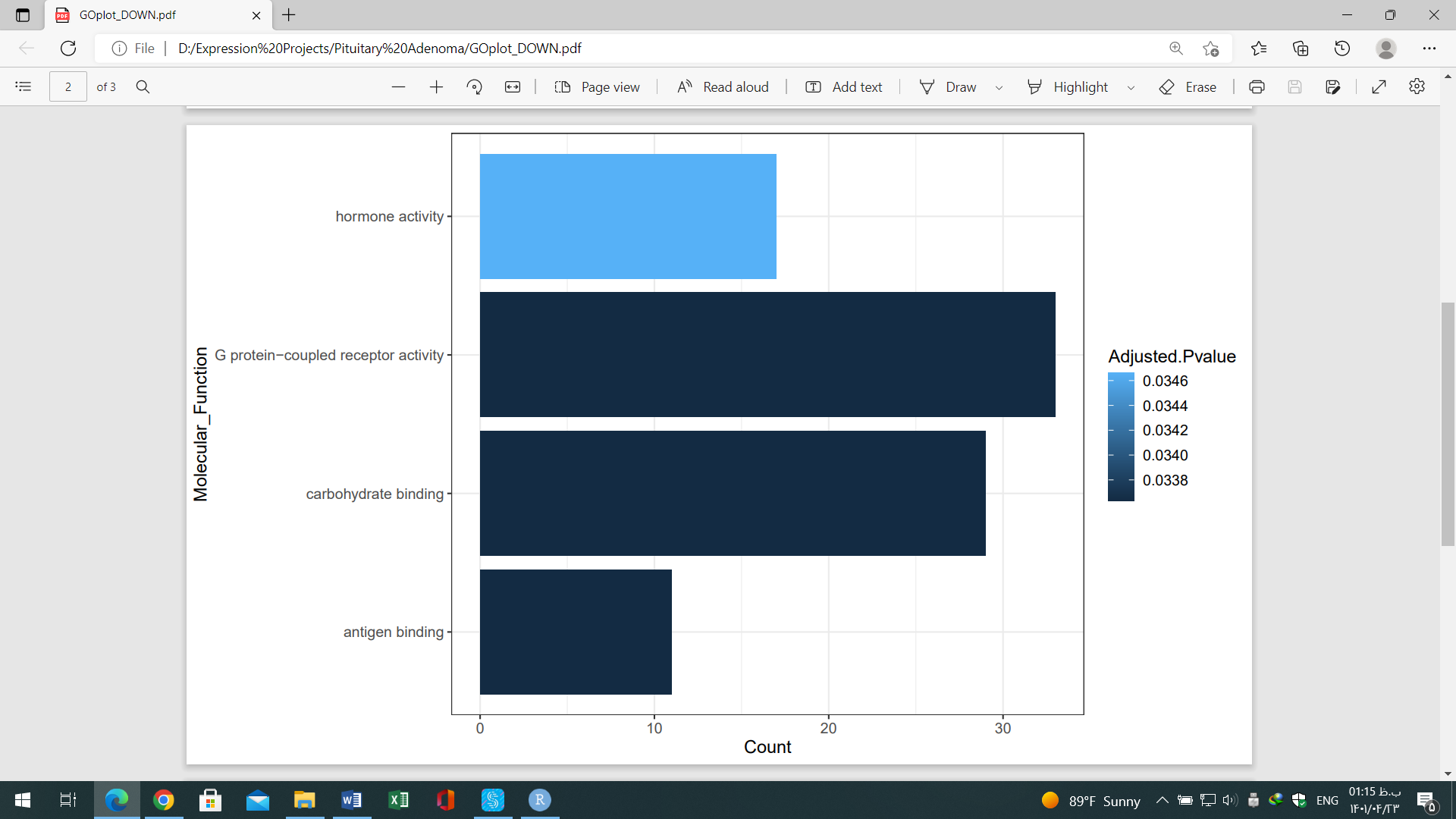


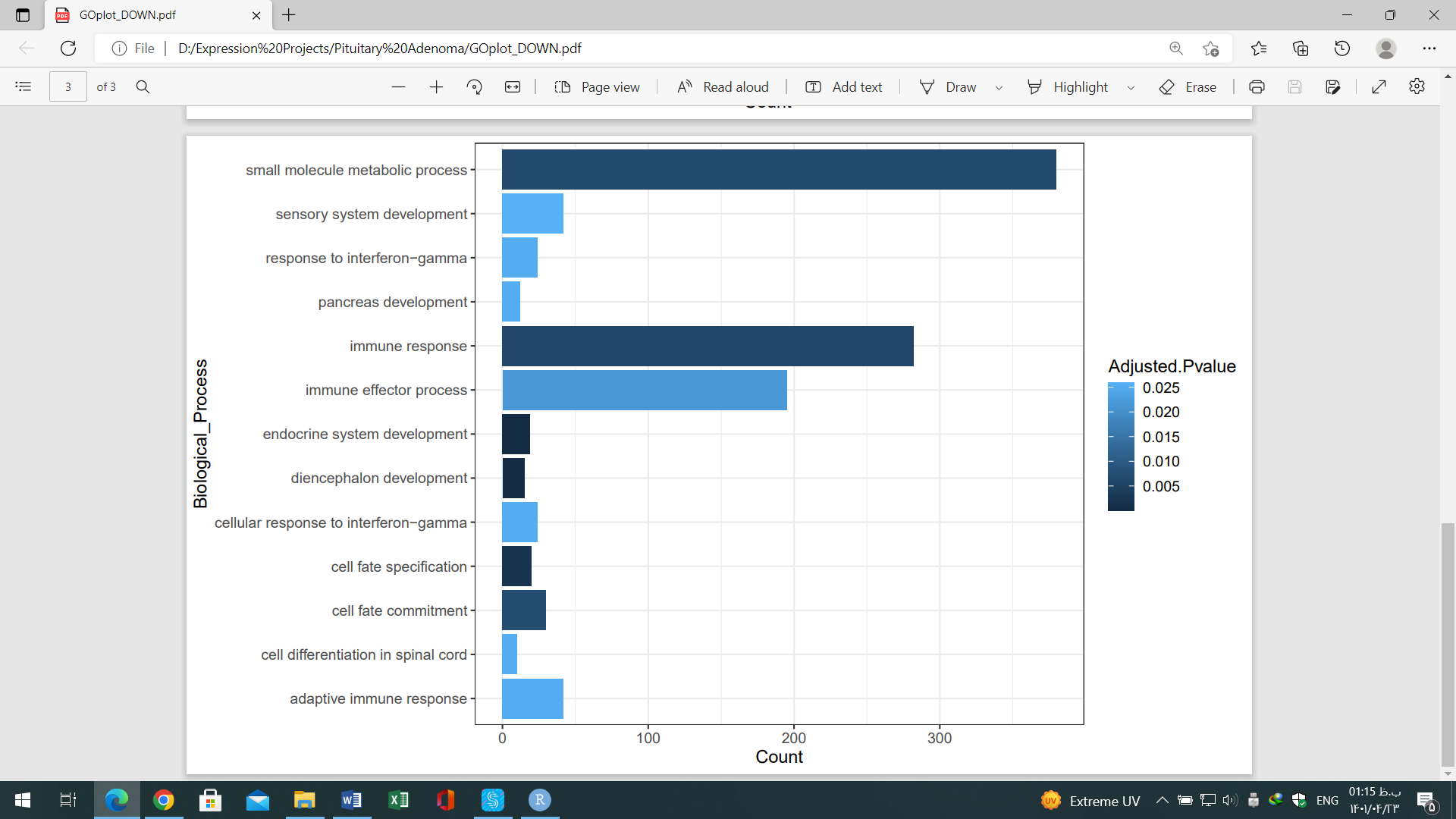


Figure S1. The barplots of function enrichment analyses. X axis shows the count of geneset; Y axis shows the geneset function; Bar color represents the adjusted P.value, ranging from dark blue (most significant) to light blue (least significant).


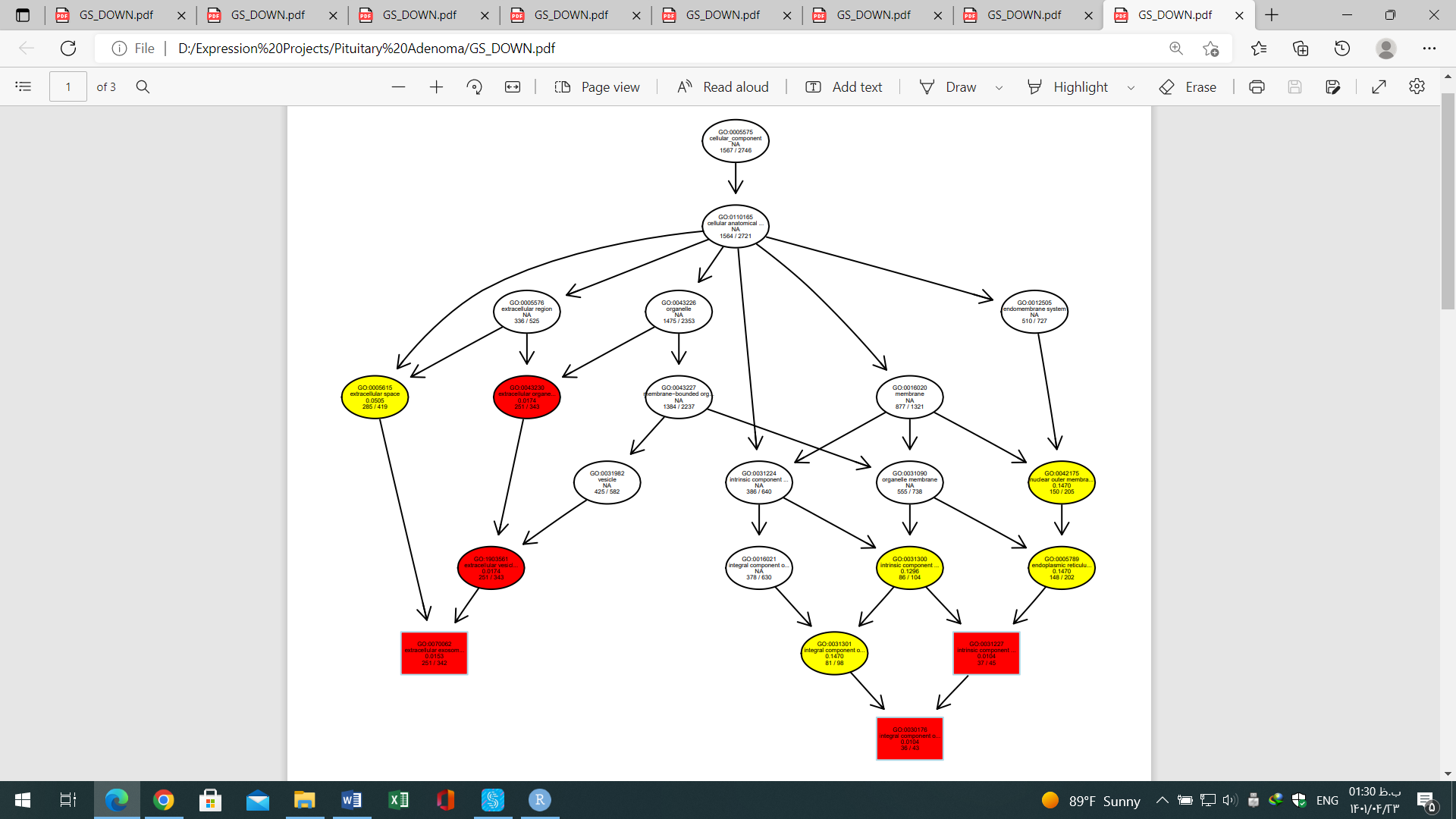


(A)


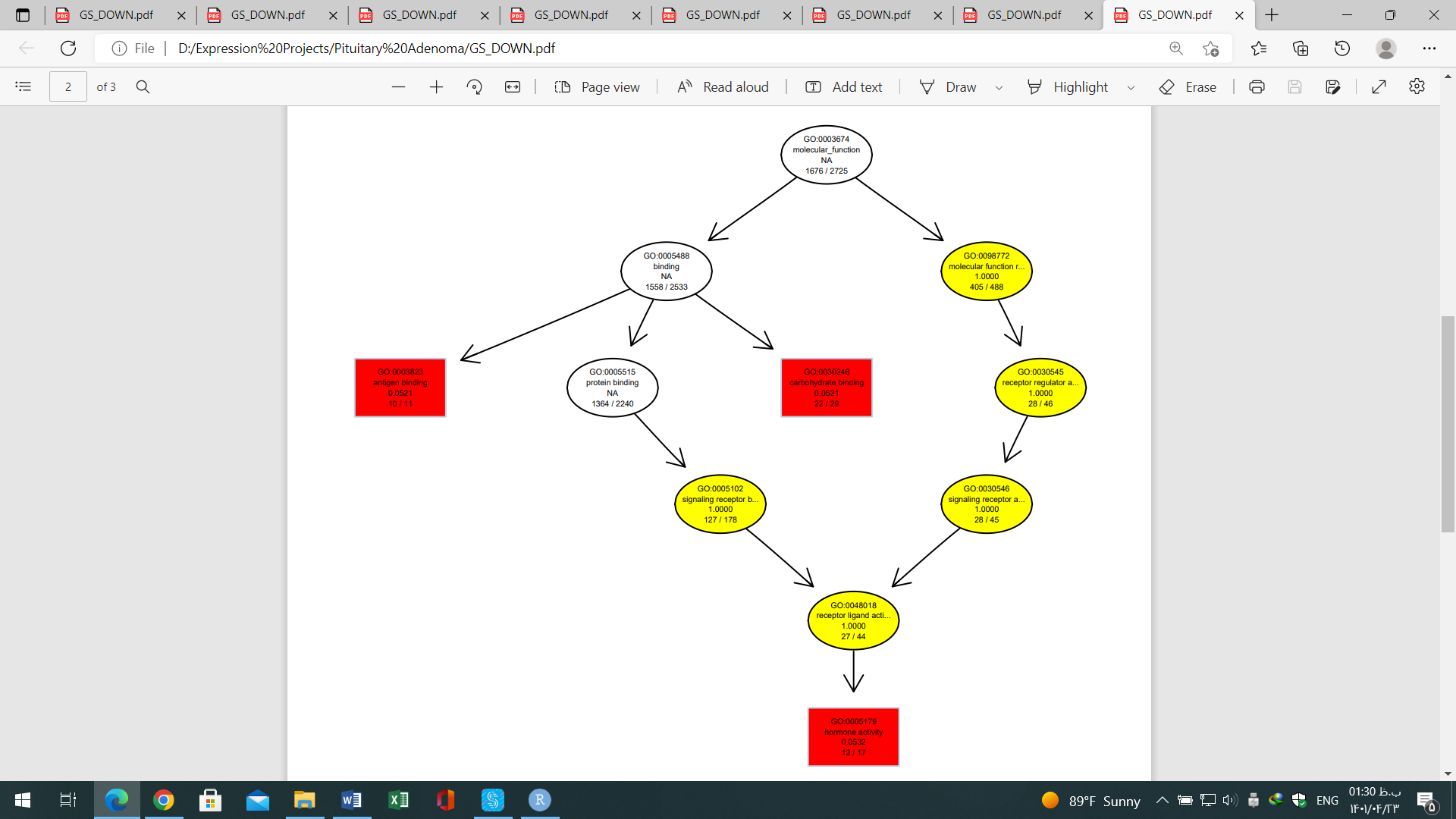


(B)


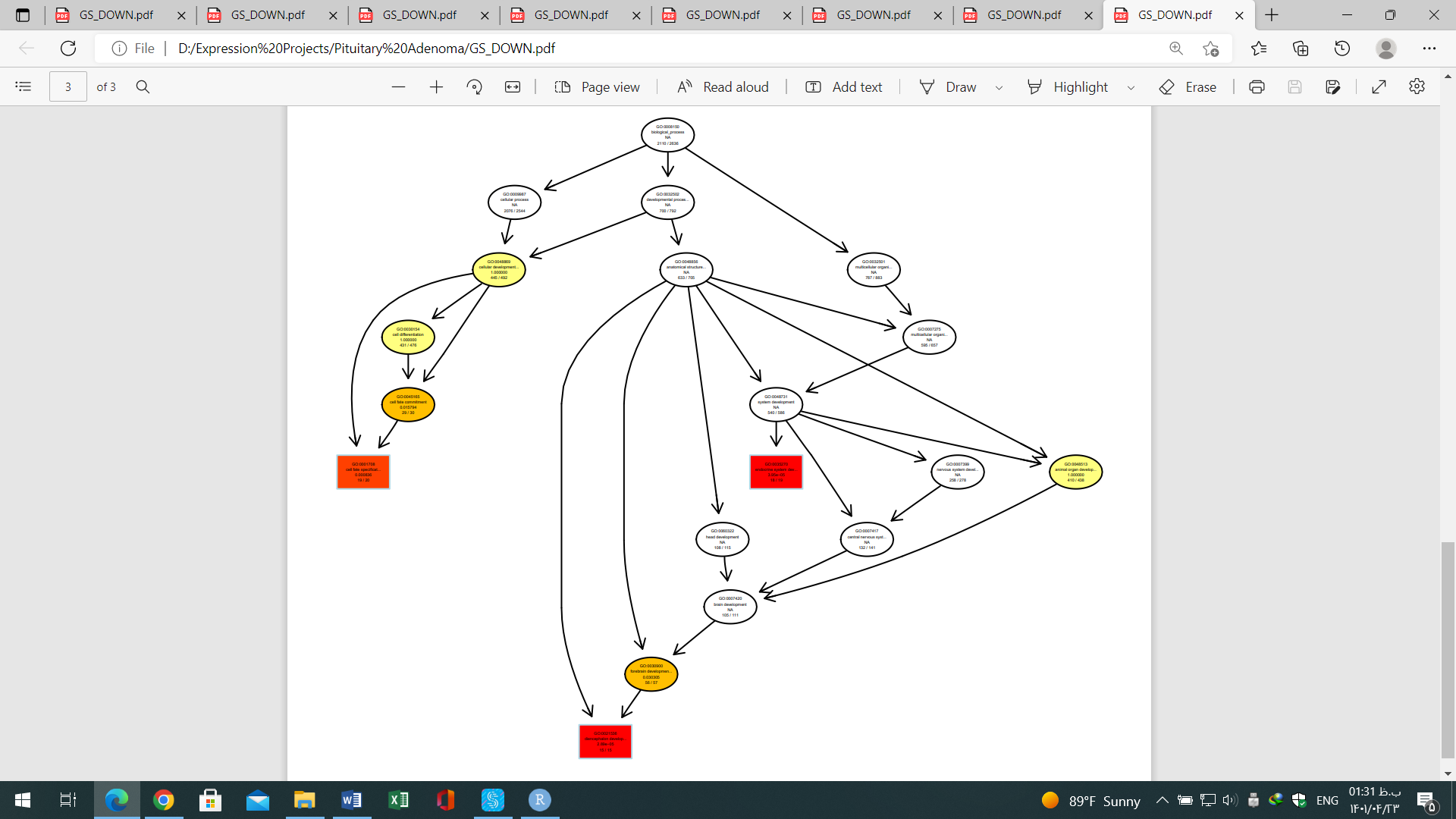


(C)

Figure S2. GO graph visualization of top GO terms enriched. (A) GO sub-graph induced by the top 3 GO terms in the category ‘Cellular Component’. (B) GO sub-graph induced by the top 3 GO terms in the category ‘Molecular Function’. (C) GO sub-graph induced by the top 3 GO terms in the category ‘Biological Process’. Boxes indicate the most significant terms. Box color represents the relative significance, ranging from dark red (most significant) to light yellow (least significant).
